# Sensory and Chemical Characteristic of Two Insect Species: *Tenebrio molitor* and *Zophobas morio* Larvae Affected by Roasting Processes

**DOI:** 10.3390/molecules26092697

**Published:** 2021-05-04

**Authors:** Anna K. Żołnierczyk, Antoni Szumny

**Affiliations:** Faculty of Biotechnology and Food Science, Wrocław University of Environmental and Life Sciences, Norwida 25, 50-375 Wrocław, Poland; antoni.szumny@upwr.edu.pl

**Keywords:** edible insects, pyrazine, Maillard reactions, aroma profile, sensory analysis

## Abstract

The volatile compounds from insects (*Tenebrio molitor* and *Zophobas morio* larvae) roasted at 160, 180, or 200 °C and fed with potato starch or blue corn flour were isolated by solid-phase microextraction (SPME), and identified by gas chromatography–mass spectrometry (GC-MS). In the tested material, 48 volatile compounds were determined. Among them, eight are pyrazines, aroma compounds that are formed in food products during thermal processing due to the Maillard reaction. Eleven of the identified compounds influenced the roast, bread, fat, and burnt aromas that are characteristic for traditional baked dishes (meat, potatoes, bread). Most of them are carbonyl compounds and pyrazines. To confirm the contribution of the most important odorants identified, their odor potential activity values (OAVs) and %OAV were calculated. The highest value was noted for isobuthylpyrazine, responsible for roast aroma (%OAV > 90% for samples roasted at lower temperatures), and 2,5-dimethylpyrazine, responsible for burnt aroma (%OAV > 20% for samples roasted at the highest temperature). According to the study, the type of feed did not significantly affect the results of the sensory analysis of roasted insects. The decisive influence was the roasting temperature. The highest scores were achieved for *Tenebrio molitor* larvae heat-treated at 160 °C.

## 1. Introduction

Insects have been eaten for over 5 million years, when our ancestors—the first hominids—consumed insects as protein supplements [1]. Some insect species are disruptive to humans and animals, for example, crop and grain storage pests [2,3]. However, we have far more benefits from the presence of insects in all the globe. Pollinating insects play a large role in nature, and eating insects can be helpful in fighting hunger in the world. At least 2 billion people in almost 80% of countries eat insects in various forms [4]. In many countries, insects are consumed because of their taste and nutritional value (they can be a source of nutritious protein, fats, and other nutrients) [5,6]. To date, more than 1900 species of insects have been described as food for humans [7,8].

In the near future, food production methods and the criteria for its choice by consumers probably will change [9], because people are increasingly aware of the need to reduce consumption and live a “zero waste” life. Production of one kilo of beef requires around ten kilograms of feed [10]. The same ten kilograms can also be a source of food for nine kilograms of insects [11]. Global meat requirements have been increasing intensively in recent years [12] and the production of “insect meat” would be a good approach for a solution to this problem. At the same time, the meat industry is one of the largest sources of pollution [13] and zoonoses such as “mad cow disease”, bird flu, or swine flu [14]. We can treat insects as an environmentally friendly animal protein in our food [15,16,17]. Insect protein is one of the four main trends (insects, vertical agriculture, aquaponics, and laboratory-grown meat) that can affect future world nutrition trends [18,19,20]. Insect meat is rich in amino acids, fats, sugars, and has a high concentration of some vitamins (e.g., B and K) [21,22].

Most Europeans do not consider insects as food, it is a food taboo [23,24,25]. Until 31 December 2017, the legal status of insects as food was unclear. Some experts believe that insects are food for general consumption, the same as farm animals. Others claimed that insects are so-called “novel food” and it is necessary to obtain the appropriate authorization [26,27]. Others thought insects were not food. In countries such as the United Kingdom, the Netherlands, or France, serving insects as a food was allowed and in Poland it was not. From January 1, 2018, insects and their parts can be placed on the market as “novel food” if they obtain the approval of the European Commission [28]. Novel food is defined as food that has not been consumed to any significant degree in the EU before 15 May 1997 (when the first novel food legislation entered into force) [29]. This can be a newly developed, innovative food or food produced using new technologies and production processes, as well as food traditionally eaten outside of the EU. For example, one of the Finnish food companies has introduced bread with the addition of insects (ground cricket powder) [30]. Insect foods can also be found in stores in Belgium, Great Britain, Denmark, and the Netherlands [31,32]. In addition, processed animal protein derived from insects may be used for feeding aquaculture animals and fur animals [33,34].

Insects are most often consumed whole (blanched, chilled, dried, fried) or ground (powdered or paste) [35,36,37]. Protein and fat isolates from insects are also used [38,39]. Known examples are protein bars with insect protein, paste with flies’ eggs, cricket flour, caterpillar burgers, larvae dumplings, crunchy fried locusts, or tempura grasshopper. The taste of insects is very diverse but it is not unusual and we can compare it to dishes known to us. It depends on the insect species and the stage of development of the insect and the method of preparation. For example, roasted grasshoppers taste like salted and oiled sardines, butter-fried locusts taste like shrimp, ant larvae have a watermelon flavor, and adult ants taste like lemon, while termites seem to taste like hazelnuts [40]. In recent years, interest in researching the biological activity of chemical compounds obtained from insects has increased. For example, peptides derived from insect proteins have anti-fungal, anti-bacterial, anti-oxidant, anti-diabetic, and antihypertensive (angiotensin-converting inhibitors (ACE)) [41,42,43].

The Maillard reactions (non-enzymatic browning reactions) were first described by Louis-Camille Maillard in 1912 [44]. It is a group of chemical reactions between amino acids and reducing sugars, usually occurring at elevated temperature, during heat treatment of food products [45]. Then, hundreds of different flavor and aroma compounds are formed in subsequent reactions. Treatment with elevated temperature causes many changes in the chemical composition and affects the nutritional value of food and its taste and smell [46]. This process creates compounds considered to be carcinogenic or mutagenic [47,48], as well as antioxidant substances with potential positive effects on the human body [49]. Some of the Maillard reaction products formed during the thermal processing of food have been known recently thanks to the development of modern separation and identification techniques [50]. Determining the chemical structures and biological properties of compounds allows improving technological processes in terms of food safety and functionality.

Therefore, the aim of the present research was to investigate the roasting of two insect species: *Tenebrio molitor* and *Zophobas morio* larvae at different temperatures (160, 180, and 200 °C) and to detect the Maillard compounds that were formed in the process. Furthermore, it was planned to see how these compounds would affect the odor profile of the roasted insects.

## 2. Results

### 2.1. Water Loss

On the basis of the obtained results (Table 1), we can conclude that during the roasting of the tested insect larvae, regardless of the temperature used and the time of thermal treatment, water is lost on average by 50%. The high water loss along with the presence of chitin in the larvae make them crunchy and not juicy after baking.

### 2.2. GC-MS Analysis

Table 2 shows the odor and flavor descriptors of 48 compounds present in the odor profile of roasted insects identified by SPME (GC-MS). The variety of these compounds is huge. There are compounds with fruit, nut, floral, vegetable, roasted, and many other aromas. Seventeen of them are carbonyl compounds (aldehydes and ketones), ten are alcohols, and eight are pyrazines, which are formed under the influence of high temperature in the baking of food products as a result of the Maillard reaction. Moreover, on the basis of literature data, odor threshold values (OTV) were assigned to the determined volatile compounds (Table 2). Identification of the compounds was done by Kovats indexes, and mass spectra of the compounds and NIST05 (NIST, 2011) spectral library collection (MS). The retention index standards used in this study consisted of a mixture of aliphatic hydrocarbons (C7–C20).

Of the 48 odorous compounds identified in roasted insects (Table 2), furan-2-carbaldehyde (1) and benzaldehyde (16) were responsible for the burnt aroma. Pyrazines: 2,5-dimethylpyrazine (7), 2-ethyl-6-methylpyrazine (24), 2-ethyl-5-methylpyrazine (25), 2,3,5-trimethylpyrazine (26), and isobutylpyrazine (34) were responsible for roast aroma in tested insects. Nonan-2-one (38) smells like milk and maltol (42) smells like baked bread. Only 11 of them have a decisive influence on the flavor of roasted insects (Table 3, Table 4, Table 5 and Table 6; complete tables in the Supplementary Material: S13 and S14).

Differences between groups of two insect species, fed in two variants (PS, BC) and roasted at three different temperatures (160, 180, and 200 °C), were determined (Table 3 and Table 4). The evaluation was based on Duncan’s test, *p* < 0.05. Based on this test, in almost all tested variants, statistical differentiation was found in the content of individual components, key for the formation of aroma of the product (the changes in compounds (16) and (24) were statistically not significant for insects fed with potato starch (Table 3)).

The potential odor activity values (OAVs) and %OAV were calculated by dividing the concentrations of aroma compounds with their sensory thresholds (OTV) from the literature. OAVs were calculated using solid phase microextraction (SPME) with a standard addition (3-ethyl-2,5-dimethylpyrazine). A calibration curve in linear range was established. Concentration ranges of the compounds were determined based on the peak areas of compounds. The highest OAV and %OAV was noted for isobutylpyrazine (**34**), responsible for roast aroma (%OAV > 90% for samples roasted at lower temperatures), and 2,5-dimethylpyrazine, responsible for burnt aroma (%OAV > 20% for samples roasted at the highest temperature). It is generally assumed that the odorants with higher OAVs contribute in a stronger manner to the overall aroma.

### 2.3. Sensory Evaluation

**Figure 1:**
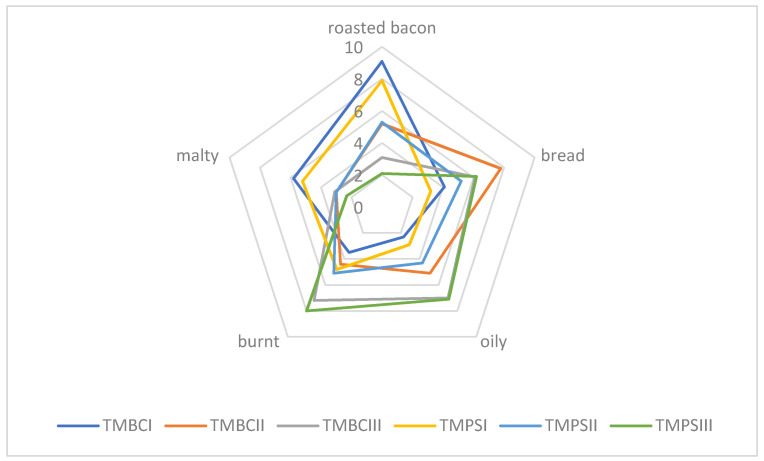
Aroma profile of the roasted *Tenebrio molitor* larvae (TMBC—*Tenebrio molitor* larvae feed blue corn flour; TMPS—*Tenebrio molitor* larvae feed potato starch).

**Figure 2:**
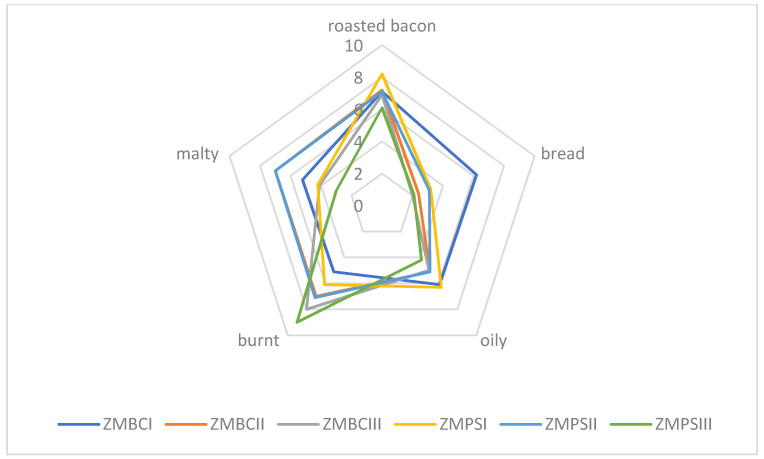
Aroma profile of the roasted *Zophobas morio* larvae (ZMBC—*Zophobas morio* larvae feed blue corn flour; ZMPS—*Zophobas morio* larvae feed potato starch).

Figure 1 and Figure 2 show a diagram for the average scores of olfactory attribute intensities of roasted larvae. Significant flavor differences were noticed between the larvae baked at different temperatures. Out of the mealworm trials in the TMBCI and TMPSI (baked in 160 °C), the dominant aromas were roasted bacon and bread. On the other hand, for the TMBCIII and TMPSIII trials, the burnt flavor turned out to be the most characteristic and they were the least acceptable in the overall assessment. The samples containing roasted superworm larvae at 160 °C were also characterized by roasted bacon and oily aroma. The larvae baked at 200 °C were burnt. The sensory analysis results are in agreement with the chemical analysis (Table 5 and Table 6). Samples roasted at 160 °C had a high value of %OAV for isobutylpyrazine (**34**), responsible for the roast aroma, while samples roasted at 200 °C had a relatively high value %OAV for 2,5-dimethylpyrazine (**7**), which was responsible for the burnt aroma, among others.

## 3. Discussion

Roasting refers to the dry thermal treatment of food in an oven and is usually applied to meat. The tested larvae *Tenebrio molitor* and *Zophobas morio* lost on average about 50% of their water when baked at three different temperatures (160, 180, and 200 °C) (Table 1). During roasting, juices (moisture) are lost and heat-labile (e.g., some vitamins are easily destroyed by heat). The study shows that the three temperatures used do not show appreciable variations in water loss. Roasting can improve the palatability and appearance of food by enhancing and preserving natural flavors. It can also improve food safety by destroying pathogenic microorganisms. It aims to increase the absorption of nutrients, allows the consumption of certain products, and gives flavor. Unfortunately, despite the overall improvement in the digestibility of, e.g., protein or carbohydrates, many ingredients are lost during thermal processing, so it is not recommended in every situation and with every product. The cooking loss is a combination of liquid and soluble substances lost from the meat during cooking. Overall, it can affect the nutritional value of food positively or negatively.

Sensory quality is one of the most important features in food, including meat products or insects, too. There are many factors affecting food quality, like, for example, feedstuff type and its composition or heat treatment. Feed components influence the nutritional and physio-chemical properties of meat and its sensory characteristics, which in turn are reflected in the quality of meat products. We assume that for insects, it will be similar. Due to the size and delicate nature of insects as food, they should be baked carefully so that they do not turn bitter and black (burnt and unpalatable). Sensory evaluation defined as “the systematic study of human reaction to physicochemical properties” enables obtaining information about the sensitivity of the human sense of taste and smell [64,65]. In sensory analysis, the respective groups were divided by the effect of feeding and temperature in the group of insect species. Standard deviation and Duncan’s test (*p* < 0.05) were applied. The corresponding Table 3, Table 4, Table 5, Table 6, Table 7 and Table 8 can be found in the Supplementary Materials S15–S20. Statistical tests show that there were in most cases significant statistical differences in all groups. The applied baking temperature had a greater effect on the smell of the insects. Mealworm larvae roasted at 160 °C (TMPSI, TMBCI) were characterized by the aroma of baked bacon, those roasted at 180 °C (TMPSII, TMBCII) showed a bread smell, and those roasted at 200 °C (TMPSIII, TMBCIII) had a burnt smell (Figure 1). In the case of superworm larvae, samples roasted at the lowest of the temperatures tested (ZMPCI, ZMBCI) were also characterized by an intense smell of roasted bacon (Figure 2). Similar results were obtained for larvae roasted at 180 °C (ZMPSII, ZMBCII), and the malty aroma was also recognized. Samples roasted at the highest temperature (ZMPSIII, ZMBCIII) were found to be burnt. Based on our own experience, we can conclude that depending on the species, we should bake insects at a temperature of 160 to 180 °C. Those temperatures are appropriate and they contribute in the panel test to major pleasantness. From the experiments carried out, the optimum temperature for baking the selected insect larvae is 160 °C (Figure 1 and Figure 2).

Among the many aromatic compounds found in food, only those that are present in a concentration greater than their sensory detection threshold are significant. The odor detection threshold and odor activity value (OAV) are important for the importance of a volatile compound to the aroma of a food product. Because most essential odorants have low odor detection thresholds, the odor of these compounds can be detected when they are at low concentrations. To estimate odor potency, the OAV is used. This is the ratio of the concentration of a volatile compound to its odor detection threshold. The relevant odor descriptors of the compounds determined by GC-MS (SPME) in roasted samples of the insect larvae studied can be divided into general groups: fruity-floral, roast, bread-like, and burnt. The results obtained in the sensory analysis are supported by the chemical analysis of the aroma compounds and in respect with their OAV values (%OAV). For insects roasted at the lowest temperature (TMPSI, TMBCI, MPSI, ZMBCI), the highest OAV was found for isobutylpyrazine (**34**) and 2,5-dimethylpyrazine (**7**) and was more than 90% and about 20%, respectively (Table 5 and Table 6). Their presence determined mainly the aroma of the roast. As the roasting temperature of the insects was increased, a burning smell developed. The isobutylpyrazine (**34**) content (%OAV) decreased (roast aroma) and the 2,5-dimethylpyrazine (**7**) content increased (burnt aroma).

Pyrazines occur naturally in heat-treated food products and are produced on a mass scale by extraction from natural products, chemical synthesis, and biocatalysis (enzymatic, microbiological) [66]. They are used as flavor- and aroma-enhancing compounds. In the case of alkyl pyrazines, the increasing demand can no longer be supplied economically from natural sources. The natural occurrence of the main seven alkyl pyrazines in foods in Europe in tons per year was 2157 tons per year (in 2004). In contrast, the annual volume of use of the same pyrazine derivatives used as flavoring agents in Europe was 2157 kg per year, which represents 0.85% of naturally occurring compounds [67]. Therefore, new, preferably natural, sources of pyrazines are being sought. Roasted insects can find use as food additives that are a source of natural pyrazines. In January 2021, specialists from the European Food Safety Authority (EFSA) gave a positive opinion on insect-based food products [68]. The project has not yet been approved by the European Commission, but specialist voices indicate that such approval is likely to be given. The novelty of using insects in food has aroused great interest among the public (potential consumers). Various insect-derived foods could be applied as a source of protein, lipids, vitamins, macro- and microelements, or volatile compounds for the diet.

## 4. Materials and Methods

### 4.1. Insect Breeding

Mealworms (TM) are the larval form of the mealworm beetle, *Tenebrio molitor*, a species of darkling beetle. Darkling beetle is the common name of the large family of beetles Tenebrionidae. In nature, it lives where it is dark, warm, humid, and there are lots of decaying organic matter, i.e., under decaying pieces of bark of deciduous trees. Moreover, *Tenebrio molitor* is a pest of grain, flour, and food stores [69]. The life cycle of *Tenebrio molitor* is of variable length, from 280 to 630 days. Larvae hatch after 10–12 days (at 18–20 °C) and become mature after a variable number of stages (8 to 20), typically after 3–4 months, but the larva stage can last up to 18 months. Mealworms have short life cycles, and are easy to breed [69].

Superworms (ZM), Morio worms, or Zophobas, *Zophobas morio* (Fabricius, 1776) (Coleoptera: Tenebrionidae) are a globally recognized feed for reptiles. In the wild, superworms larvae occur in dead (diseased) trees, where they feed on this wood. It is a popular food insect due to its ease of breeding and nutrition. Recent research efforts indicate that this insect could also be used as a partial replacement of fishmeal for farmed tilapia [70]. The life cycle of *Z. morio* is like other beetles, as it has an egg, larva, pupa, and adult stage. Larvae are similar to mealworm larvae, although they are much larger and more fat.

Insects for described research were purchased at a local terrarium store (Wrocław, Poland). The feeding medium for the insect larvae consisted of 100% blue corn flour (BC) or potato starch (PS). Rearing of larvae was carried out in plastic containers (in triplicate for each of the food variants) at 26 °C for 10 days. For 500 g of insect biomass, 500 g of blue corn flour (BC) and potato starch (PS) were added, respectively. After 5 days, another 200 g of feed was added.

At the end of the ten-day growth period, the larvae were separated from the feeding media by manual sieving and immediately preserved by freezing at −28 °C. The larvae were weighed about 1 g into separate glass screw-cap containers and stored at −28 °C until analysis. The mean larval weight for mealworms was 0.13 g and for superworms was 0.51 g. The larvae were baked whole and were crushed before SPME analysis.

### 4.2. Insect Samples

On the day of the analysis of the profile of fragrances (SPME), insects were thawed to room temperature and then baked according to the three following variants: I. 160 °C for 20 min; II. 180 °C for 15 min; III. 200 °C for 10 min (Table 7). After the baking process, the dishes were sealed and the samples were prepared for SPME analysis (in sub-replicate, three times).

### 4.3. Water Loss (W_L_)

Non-enzymatic browning reactions are not only chemically complicated. Physical phenomena also have an impact on Maillard’s reaction. One of the factors conditioning the reaction of non-enzymatic browning during heat treatment food is water activity (aw). In the tested materials, water loss during baking was expressed as g/100 g and was calculated by weighing the insect samples before (W_B_) and after roasting (W_A_), as follows: W_L_ =100 × (W_B_ − W_A_) / W_B_. The determination of each variant was carried out in triplicate.

### 4.4. SPME/GC-MS Conditions

For HS-SPME analysis (30 min exposure to a 2 cm DVB/CAR/PDMS fiber (Supelco, Bellefonte, PA, USA)), about 0.5 g of roasted sample was put in to headspace vials and kept in a laboratory water bath at 50 °C. Next, 0.1 µg of equilibrium mixture of 3-ethyl-2,5-dimethylpyrazine and 2-ethyl-3,5-dimethylpyrazines (Sigma Aldrich, Saint Louis, MO, USA) as an internal standard was added. Calibration function was constructed for 3-ethyl-2,5-dimethylpyrazine and 2-ethyl-3,5-dimethylpyrazines ranging from 0.001 to 1 microgram (in vial suspended and intensively shaken in water before use), with excellent linearity, with an R^2^ value 0.993. We observed two signals with equal ratio. Semi-quantification of compounds was based on calculation of the area of unknown signals and comparison with the regression equation for the internal standard.

Analyte desorption (220 °C for 3 min) was performed on Shimadzu apparatus (Shimadzu, Kyoto, Japan) equipped with a Zebron ZB-5 MSI (30 m × 0.25 mm × 0.25 µm) column (Phenomenex, Shim-Pol, Warsaw, Poland). Fiber composition was chosen due to previous optimizations [71]. The potential OAV was calculated by dividing the concentration of the compounds in the sample by the sensory thresholds obtained from the literature. The concentration of the compounds was established by a standard calibration curve. The Kovats retention index values were calculated for each according to Adams [72], with a comparison of the obtained data with the values presented in NIST17 (NIST/EPA/NIH Mass Spectral Library) database peaks by comparing their retention characteristics with those of the two closest eluting aliphatic hydrocarbons from the retention index standard, analyzed under identical conditions. Presumptive identification can often be made by comparing the Kovats retention index value with a value previously published in literature references. Identification of the compounds was done by comparison: I: spectrum presented in NIST17 (NIST/EPA/NIH Mass Spectral Library); II: calculated retention index values with database NIST17; III: retention times of unknown compounds with available standards (1, 3, 5, 8, 11, 14, 15, 16, 18, 20, 23, 30, 32, 39, 40, 43, 44, 45, 47, 48).

### 4.5. Sensory Evaluation

In this study, for sensory evaluation, we chose descriptive sensory analysis. A nine-hedonic scale (Table 8) was used to investigate the degree of preference of the roasted larvae (160, 180, and 200 °C by 20, 15, or 10 min, respectively) of *Tenebrio molitor* (TM) and *Zophobas morio* (ZM) fed with potato starch (PS) or blue corn flour (BC) (sample codes in Table 7). Samples were roasted and 250 g of each sample were used. After thermal treatment, the insects were put in glass containers and stored in a fridge (−24 °C) until sensory and GC-MS analysis. Nine panelists were chosen from the teaching staff, graduate students, and master degree students of The Faculty of Biotechnology and Food Science, Wrocław University of Environmental and Life Sciences. The age distribution of the panelists was between 23 and 48. Among them were four men and five women. The panelists were trained for three one-hour sessions. To assist the panelists in establishing a framework for each attribute, reference smells were used during training to establish minimum and maximum intensities for each attribute. The samples were evaluated for sensory quality of roasted bacon, bread, oily, burnt, and malty aroma, and consistency using a varying scale from 9—which means like extremely, to 1—which means dislike extremely (Table 8). Descriptors for the evaluation of roasted insects were designated from the literature data about roasted food and in preliminary tests. A set of reference solutions in water (0.01–0.1%; concentrations well above the threshold, but assessed as not very intense) was prepared based on the odor descriptor set, which consisted of coffee (no. 12), bread crust (no. 85), bread (no. 86), beef found (no. 178), pork found (no. 179 (Sosa Ingredients, S.L., Spain)), malty aroma (soya milk, Mona Naturprodukte GmbH, Austria) and caramel (burned sugar). The sensory tests were done in a specially designed laboratory, which met relevant standards. The three treatments were evaluated in one session. The data were recorded on paper. The samples were coded and randomized. The insect samples were served on small plates. After each sample, the panelists drank water to restore their original tasting conditions.

### 4.6. Statistical Analysis

The data from quantitative volatile constituents were subjected to the analysis of variance using Duncan’s test (*p* < 0.05), all using the STATISTICA 13.3 software for Windows (StatSoft, Krakow, Poland).

## 5. Conclusions

There are clear environmental, economic, and nutritional aspects of breeding insects for feed and food. Moreover, the smell, taste, colour, and texture of a meal determines its acceptability. The sense of smell allows an initial evaluation of the taste we can expect, and reinforces its sensation when the food is in the mouth. During chewing and swallowing, the aroma reaches the inner part of the nose and this helps to enhance the culinary experience. Pyrazines and carbonyl compounds (Maillard reaction products) formed during the thermal treatment of insects determine the sensation of odor. It is therefore important to choose the right roasting conditions. Based on the performed sensory analysis and GC-MS (SPME), the characteristic aroma of roasted insects is shaped by 11 odor-active compounds. The %OAV of pyrazines for 160 °C roasted larvae was over 99% for mealworms and superworms. This is supposed to determine the strong roasted flavor in these samples. Mealworms roasted at 180 °C were characterized by a pleasant and desirable bread smell. In contrast, mealworms roasted at the highest temperature tested (200 °C) were characterized by an undesirable burnt smell.

## Figures and Tables

**Table 1:** Water loss (W_L_) in insects during roasting.

Insect Roasting Variants	W_L_	Insect Roasting Variants	W_L_
TMBCI	55.0 ± 1.8 b	ZMBCI	48.5 ± 1.1 c
TMBCII	54.6 ± 0.6 b,c	ZMBCII	48.7 ± 0.8 c
TMBCIII	57.1 ± 0.9 a	ZMBCIII	48.3 ± 0.3 c
TMPSI	57.3 ± 4.3 a	ZMPSI	51.3 ± 1.2 a
TMPSII	51.6 ± 0.8 d	ZMPSII	50.7 ± 0.3 b
TMPSIII	49.8 ± 1.0 e	ZMPSIII	51.2 ± 0.9 a

Mean values with different letters (a–e) within the same column were statistically different (*p* < 0.05), the same letters form one homogeneous group. Values expressed as mean ± standard deviation.

**Table 2:** Description of odor and odor threshold values (OTV) of odor-active compounds in roasted insects. Literature Kovats indexes were obtained from NIST database (2017).

No	Compound	Kovats Indexes		OTV [ppm]	Odor Description
		Exp.	Lit.		
1	Furan-2-carbaldehyde	843	845	0.5	Almond, baked potatoes, bread, burnt, spice [51]
2	5-Methylhex-5-en-2-one	866	873	−	Green
3	Heptan-2-one	894	898	0.6	Intense fruity
4	(*E*)-5-methylhex-3-en-2-one	901	896	−	Fruity
5	heptanal	903	906	0.021	Citrus, fat, green, nut
6	3-methylsulfanylpropanal	907	909	0.0002	Cooked potato, soy
7	2,5-Dimethylpyrazine	911	912	0.1	Cocoa, roast beef, roasted nut, burnt
8	2-Acetylfuran	912	913	10	Balsamic, cocoa, coffee
9	2-Ethylpyrazine	914	916	100	Burnt, green, must, peanut butter, roasted, rum, wood
10	2,3-Dimethylpyrazine	919	916	20	Caramel, cocoa, hazelnut, peanut butter, roasted
11	α-Pinene	933	936	4	Cedarwood, pine, sharp
12	2-Ethyl-1*H*-pyrrole	947	941	0.06	[52]
13	2,5-Dimethylfuran-3-one	948	948	0.006	Fruity estery, caramellic
14	Linalool	952	949	0.01	Coriander, floral, lavender, lemon, rose
15	Heptan-2-ol	956	960	0.8	Citrus, earth, fried, mushroom, oil
16	Benzaldehyde	960	962	0.1	Bitter almond, burnt sugar, cherry, malt, roasted, pepper
17	2-Ethyl-4-methyl-1,3-thiazole	965	971	0.00001	Savory
18	Dimethyl trisulfide	972	969	0.06	Cabbage, sish, onion, sulfur
19	Seudenol	977	977		Pheromone [53]
20	Oct-1-en-3-ol	983	978	0.05	Cucumber, earth, fat, floral, mushroom
21	Sulcatone	989	986	0.05	Citrus, mushroom, pepper, rubber, strawberry
22	Yomogi alcohol	995	996	−	Greenish
23	Octan-2-ol	998	1003	0.1	Fat, mushroom
24	2-Ethyl-6-methylpyrazine	1001	1000	0.1	Roasted hazelnut [54], buckwheat tea [55], fruity and flowery in coffee [56], fruity in roasted sesame [57], almond and nutty in wild rice [58]
25	2-Ethyl-5-methylpyrazine	1002	1004	15	Fruit, green
26	2,3,5-Trimethylpyrazine	1004	1005	0.1	Cocoa, earth, must, potato, roast
27	2-Ethyl-3-methylpyrazine	1007	1006	0.12	Green, must, nut, potato, roast
28	Pseudocumene	1019	1024	−	Plastic [59]
29	2-Acetylthiazole	1021	1021	0.01	Nut, popcorn, roast, sulfur
30	Limonene	1030	1032	0.01	Purgent, lemon-like [60], sour [61]
31	Eucalyptol	1035	1032	0.012	Camphor, cool, eucalyptol, mint
32	2-Phenylacetaldehyde	1045	1046	4	Berry, geranium, honey, nut, pungent
33	Oct-2-enal	1050	1059	0.003	Dandelion, fat, fruit, grass, green, spice
34	Isobutylpyrazine	1056	1060	0.000016	Green, pepper, coffee, roasted
35	Oct-3-en-1-ol	1061	1060	0.0012	Dust, toasted nut
36	2-acetyl pyrrole	1064	1074	0.2	Bread, cocoa, hazelnut, licorice, walnut
37	Oct-2-en-1-ol	1067	1067	0.84	Green, citrus, vegetable, fatty
38	Nonan-2-one	1096	1093	0.1	Fragrant, fruit, green, hot milk
39	Undecane	1100	1100	56	Gasoline-like
40	Nonanal	1108	1107	40	Fat, green, lemon
41	(*E*)-5-Methyl-2-propan-2-ylhex-2-enal	1112	1109		Floral
42	Maltol	1116	1108	0.035	Sweet, caramel-like, cotton, candy, fruity, bread, baked
43	*p*-Menthatriene	1119	1110	0.6	Oily, chemical, cooling, woody, pine, thyme, herbal, tropical
44	Dodecane	1200	1200	0.766	Gasoline-like
45	Decanal	1208	1208	0.02	Floral, fried, orange peel, penetrating, tallow
46	2-Decen-1-ol	1268	1270	−	Fruit
47	Tridecane	1300	1300	−	Gasoline-like
48	Tetradecane	1400	1400	−	Gasoline-like

Odor descriptors for individual compounds were taken from the Pubchem [62] and The Good Scents Company Information System [63] or from the scientific articles.

**Table 3:** Concentration of aroma active compounds of roasted *Tenebrio molitor* and *Zophobas morio* larvae fed with potato starch (PS).

No	Compound	TMPSI	TMPSII	TMPSIII	ZMPSI	ZMPSII	ZMPSIII
1	Furan-2-carbaldehyde	n.d.	n.d.	0.745 ± 0.096 A	n.d.	n.d.	n.d.
7	2,5-Dimethylpyrazine	n.d.	0.044 ± 0.005 B	0.049 ± 0.006 B	0.011 ± 0.002 D	0.028 ± 0.005 C	0.457 ± 0.037 A
16	Benzaldehyde	n.d.	0.006 ± 0.003 A	0.002 ± 0.001 A	0.004 ± 0.002 A	0.003 ± 0.001 A	0.006 ± 0.002 A
24	2-Ethyl-6-methylpyrazine	n.d.	0.012 ± 0.003 A	n.d.	0.014 ± 0.001 A	0.012 ± 0.001 A	n.d.
25	2-Ethyl-5-methylpyrazine	n.d.	n.d.	0.058 ± 0.004 A	n.d.	n.d.	0.037 ± 0.004 B
26	2,3,5-Trimethylpyrazine	0.031 ± 0.011 B	0.013 ± 0.004 D	0.048 ± 0.003 A	0.021 ± 0.003 C	0.014 ± 0.002 D	0.027 ± 0.011 C
27	2-Ethyl-3-methylpyrazine	n.d.	n.d.	n.d.	0.004 ± 0.002 A	0.014 ± 0.003 B	n.d.
34	Isobutylpyrazine	0.054 ± 0.008 A	0.003 ± 0.002 C	0.017 ± 0.002 B	0.003 ± 0.001 C	0.0003 ± 0.0001 D	0.001 ± 0.0005 C
35	Oct-2-en-1-ol	0.027 ± 0.009 A	0.003 ± 0.001 C	0.012 ± 0.002 B	0.002 ± 0.001 C	n.d.	n.d.
38	Nonan-2-one	0.025 ± 0.005 A	0.003 ± 0.002 B	n.d.	n.d.	0.003 ± 0.001 B	n.d.
42	Maltol	0.108 ± 0.012 A	0.004 ± 0.001 C	0.032 ± 0.004 B	0.022 ± 0.004 B	0.004 ± 0.002 C	0.036 ± 0.007 B

Mean values with different letters (A–D) within the same row were statistically different (*p* < 0.05), the same letters form one homogeneous group. Values expressed as mean ± standard deviation. n.d. = not determined.

**Table 4:** Concentration of aroma active compounds of roasted *Tenebrio molitor* and *Zophobas morio* larvae fed with blue corn flour (BC).

No	Compound	TMBCI	TMBCII	TMBCIII	ZMBCI	ZMBCII	ZMBCIII
1	Furan-2-carbaldehyde	n.d.	n.d.	0.055±0.008 A	n.d.	n.d.	n.d.
7	2,5-Dimethylpyrazine	0.098 ± 0.007 B	0.437 ± 0.011 A	0.049 ± 0.003 D	0.030 ± 0.005 E	0.052 ± 0.006 C	0.063 ± 0.027 C
16	Benzaldehyde	n.d.	0.076 ± 0.0030 C	0.005 ± 0.003 D	0.004 ± 0.001 D	0.825 ± 0.073 A	0.655 ± 0.89 B
24	2-Ethyl-6-methylpyrazine	n.d.	0.167 ± 0.012 A	n.d.	0.013 ± 0.003 B	0.008 ± 0.001 B	n.d.
25	2-Ethyl-5-methylpyrazine	n.d.	n.d.	0.063 ± 0.009 A	n.d.	0.009 ± 0.003 C	0.047 ± 0.09 B
26	2,3,5-Trimethylpyrazine	n.d.	0.189 ± 0.013 B	0.046 ± 0.005 C	0.013 ± 0.005 D	0.009 ± 0.003 D	0.036 ± 0.007 C
27	2-Ethyl-3-methylpyrazine	n.d.	0.095 ± 0.008 A	n.d.	0.002 ± 0.001 B,C	0.004 ± 0.003 B,C	0.011 ± 0.002 B
34	Isobutylpyrazine	n.d.	0.033 ± 0.007 A	0.011 ± 0.004 B	0.0002 ± 0.0001 C	0.0001 ± 0.00007 C	0.00005 ± 0.00002 D
35	Oct-2-en-1-ol	n.d.	0.048 ± 0.006 A	0.011 ± 0.002 B	n.d.	n.d.	n.d.
38	Nonan-2-one	0.043±0.003 A	0.022 ± 0.003 B	0.006 ± 0.003 C	0.002 ± 0.001 C	0.008 ± 0.002 C	n.d.
42	Maltol	n.d.	0.022 ± 0.002 A	n.d.	0.005 ± 0.002 B	n.d.	n.d.

Mean values with different letters (A–E) within the same row were statistically different (*p* < 0.05), the same letters form one homogeneous group. Values expressed as mean ± standard deviation. n.d. = not determined.

**Table 5:** %OAV of roasted *Tenebrio molitor* larvae.

No	Compound	TMPSI	TMPSII	TMPSIII	TMBCI	TMBCII	TMBCIII
1	Furan-2-carbaldehyde	−	−	4.30	−	−	1.65
7	2,5-Dimethylpyrazine	−	0.14	3.52	69.56	0.20	10.88
16	Benzaldehyde	−	42.59	29.67	−	0.49	23.37
24	2-Ethyl-6-methylpyrazine	−	0.04	−	−	0.08	−
25	2-Ethyl-5-methylpyrazine	−	−	0.00	−	−	0.00
26	2,3,5-Trimethylpyrazine	0.01	0.04	0.03	−	0.09	0.07
27	2-Ethyl-3-methylpyrazine	−	−	−	−	0.037	−
34	Isobutylpyrazine	99.24	56.38	61.86	−	97.22	62.64
35	Oct-2-en-1-ol	0.66	0.76	0.57	−	1.85	1.39
38	Nonan-2-one	0.01	0.01	−	30.44	0.01	0.01
42	Maltol	0.09	0.04	0.05	−	0.03	−

**Table 6:** %OAV of roasted *Zophobas morio* larvae.

No	Compound	TMPSI	TMPSII	TMPSIII	TMBCI	TMBCII	TMBCIII
1	Furan-2-carbaldehyde	−	−	−	−	−	−
7	2,5-Dimethylpyrazine	0.05	1.35	6.53	2.03	6.01	11.79
16	Benzaldehyde	0.002	0.02	0.01	0.03	11.89	15.39
24	2-Ethyl-6-methylpyrazine	0.06	0.57	−	0.85	0.97	−
25	2-Ethyl-5-methylpyrazine	−	−	0.003	−	0.01	0.06
26	2,3,5-Trimethylpyrazine	0.09	0.65	0.39	0.86	0.99	6.80
27	2-Ethyl-3-methylpyrazine	0.02	0.54	−	0.14	0.35	1.65
34	Isobutylpyrazine	98.76	96.17	91.60	94.95	78.83	64.32
35	Oct-2-en-1-ol	0.73	−	−	−	−	−
38	Nonan-2-one	−	0.15	−	0.12	0.95	−
42	Maltol	0.28	0.55	1.47	1.02	−	−

**Table 7:** Roasting condition and sample codes.

Insect	Feed	Roasting Temperature [°C]	Roasting Time [min]	Sample Code
*Tenebrio molitor*	Blue corn flour	160	20	TMBCI
		180	15	TMBCII
		200	10	TMBCIII
	Potato starch	160	20	TMPSI
		180	15	TMPSII
		200	10	TMPSIII
*Zophobas morio*	Blue corn flour	160	20	ZMBCI
		180	15	ZMBCII
		200	10	ZMBCIII
	Potato starch	160	20	ZMPSI
		180	15	ZMPSII
		200	10	ZMPSIII

**Table 8:** Nine-point hedonic scale was used in the preference test.

Grade	Score
Like extremely	9
Like very much	8
Like moderately	7
Like slightly	6
Neither like or dislike	5
Dislike slightly	4
Dislike moderately	3
Dislike very much	2
Dislike extremely	1

## Data Availability

Data is contained within the article or Supplementary Material.

## Supplementary Materials

The following are available online, S1: Chromatogram, TMBCI; S2: Chromatogram, TMBCII; S3: Chromatogram, TMBCIII; S4: Chromatogram, ZMBCI; S5: Chromatogram, ZMBCII; S6: Chromatogram, ZMBCIII; S7: Chromatogram, TMPSI; S8: Chromatogram, TMPSII; S9: Chromatogram, TMPSIII; S10: Chromatogram, ZMPSI; S11: Chromatogram, ZMPSII; S12: Chromatogram, ZMPSIII; S13: Table S1. Concentration and odor activity values of aroma active compounds (OAV) and %OAV of roasted *Tenebrio molitor* larvae; S14: Table S2. Concentration and odor activity values of aroma active compounds (OAV) and %OAV of roasted *Zophobas morio* larvae; S15: Table S3. Aroma profile of the roasted at 160 °C *Tenebrio molitor* larvae fed BC or PS; S16: Table S4. Aroma profile of the roasted at 180 °C *Tenebrio molitor* larvae fed BC or PS; S17: Table S5. Aroma profile of the roasted at 200 °C *Tenebrio molitor* larvae fed BC or PS; S18: Table S6. Aroma profile of the roasted at 160 °C *Zophobas morio* larvae fed BC or PS; S19: Table S7. Aroma profile of the roasted at 180 °C *Zophobas morio* larvae fed BC or PS; S20: Table S8. Aroma profile of the roasted at 200 °C *Zophobas morio* larvae fed BC or PS.

## Sample Availability

Samples of the compounds are not available from the authors.

## References

[1] Lesnik J.J. (2014). Termites in the hominin diet: A meta-analysis of termite genera, species and castes as a dietary supplement for South African robust australopithecines. J. Hum. Evol..

[2] Kent R.B. (1989). The African honeybee in Peru: An insect invader and its impact on beekeeping. Appl. Geogr..

[3] Midega C.A., Murage A.W., Pittchar J.O., Khan Z.R. (2016). Managing storage pests of maize: Farmers’ knowledge, perceptions and practices in western Kenya. Crop Prot..

[4] Tao J., Li Y.O. (2018). Edible insects as a means to address global malnutrition and food insecurity issues. Food Qual. Saf..

[5] Ramos-Elorduy J. (1997). Insects: A sustainable source of food?. Ecol. Food Nutr..

[6] Ramos-Elorduy J. (2005). Insects: A hopeful food source. Ecol. Implic. Minilivestock.

[7] Van Huis A. (2013). Potential of insects as food and feed in assuring food security. Annu. Rev. Entomol..

[8] Pal P., Roy S. (2014). Edible insects: Future of human food—A review. Int. Lett. Nat. Sci..

[9] Menozzi D., Sogari G., Veneziani M., Simoni E., Mora C. (2017). Eating novel foods: An application of the Theory of Planned Behaviour to predict the consumption of an insect-based product. Food Qual. Prefer..

[10] Smil V. (2002). Nitrogen and food production: Proteins for human diets. Ambio A J. Hum. Environ..

[11] Wegier A., Alavez V., Pérez-López J., Calzada L., Cerritos R. (2018). Beef or grasshopper hamburgers: The ecological implications of choosing one over the other. Basic Appl. Ecol..

[12] Tilman D., Cassman K.G., Matson P.A., Naylor R., Polasky S. (2002). Agricultural sustainability and intensive production practices. Nature.

[13] Jayathilakan K., Sultana K., Radhakrishna K., Bawa A. (2012). Utilization of byproducts and waste materials from meat, poultry and fish processing industries: A review. J. Food Sci. Technol..

[14] Greger M. (2007). The human/animal interface: Emergence and resurgence of zoonotic infectious diseases. Crit. Rev. Microbiol..

[15] Oonincx D.G., Van Itterbeeck J., Heetkamp M.J., Van Den Brand H., Van Loon J.J., Van Huis A. (2010). An exploration on greenhouse gas and ammonia production by insect species suitable for animal or human consumption. PLoS ONE.

[16] Sánchez-Muros M.-J., Barroso F.G., Manzano-Agugliaro F. (2014). Insect meal as renewable source of food for animal feeding: A review. J. Clean. Prod..

[17] Halloran A., Roos N., Eilenberg J., Cerutti A., Bruun S. (2016). Life cycle assessment of edible insects for food protein: A review. Agron. Sustain. Dev..

[18] Alexander P., Brown C., Arneth A., Dias C., Finnigan J., Moran D., Rounsevell M.D. (2017). Could consumption of insects, cultured meat or imitation meat reduce global agricultural land use?. Glob. Food Secur..

[19] Goddek S., Joyce A., Kotzen B., Dos-Santos M. (2019). Aquaponics and global food challenges. Aquaponics Food Production Systems.

[20] Germer J., Sauerborn J., Asch F., de Boer J., Schreiber J., Weber G., Müller J. (2011). Skyfarming an ecological innovation to enhance global food security. J. Verbrauch. Lebensm..

[21] Żołnierczyk A.K. (2019). Nutritional Properties of Edible Insects. Environmental, Health, and Business Opportunities in the New Meat Alternatives Market.

[22] Rumpold B.A., Schlüter O.K. (2013). Potential and challenges of insects as an innovative source for food and feed production. Innov. Food Sci. Emerg. Technol..

[23] Shelomi M. (2015). Why we still don’t eat insects: Assessing entomophagy promotion through a diffusion of innovations framework. Trends Food Sci. Technol..

[24] Mancini S., Moruzzo R., Riccioli F., Paci G. (2019). European consumers’ readiness to adopt insects as food. A review. Food Res. Int..

[25] Sogari G., Menozzi D., Mora C. (2019). The food neophobia scale and young adults’ intention to eat insect products. Int. J. Consum. Stud..

[26] Belluco S., Halloran A., Ricci A. (2017). New protein sources and food legislation: The case of edible insects and EU law. Food Secur..

[27] Lähteenmäki-Uutela A., Grmelová N. (2016). European law on insects in food and feed. Eur. Food Feed Law Rev..

[28] Turck D., Bresson J.L., Burlingame B., Dean T., Fairweather-Tait S., Heinonen M., Hirsch-Ernst K.I., Mangelsdorf I., McArdle H., EFSA Panel on Dietetic Products, Nutrition and Allergies (NDA) (2016). Guidance on the preparation and presentation of an application for authorisation of a novel food in the context of Regulation (EU) 2015/2283. Efsa J..

[29] Regulation H.A.T. (1997). Regulation (EC) No 258/97 of the European Parliament and of the Council of 27 January 1997 concerning novel foods and novel food ingredients. Off. J. Eur. Communities.

[30] Chen Y. (2018). Potential of Industrial Side Streams in Insect Production. https://www.theseus.fi/handle/10024/156689.

[31] House J. (2016). Consumer acceptance of insect-based foods in the Netherlands: Academic and commercial implications. Appetite.

[32] Van Thielen L., Vermuyten S., Storms B., Rumpold B., Van Campenhout L. (2019). Consumer acceptance of foods containing edible insects in Belgium two years after their introduction to the market. J. Insects Food Feed.

[33] Makkar H.P., Tran G., Heuzé V., Ankers P. (2014). State-of-the-art on use of insects as animal feed. Anim. Feed Sci. Technol..

[34] Verbeke W., Spranghers T., De Clercq P., De Smet S., Sas B., Eeckhout M. (2015). Insects in animal feed: Acceptance and its determinants among farmers, agriculture sector stakeholders and citizens. Anim. Feed Sci. Technol..

[35] Mitsuhashi J. (1997). Insects as traditional foods in Japan. Ecol Food Nutr.

[36] Chen X., Feng Y., Chen Z. (2009). Common edible insects and their utilization in China. Entomol. Res..

[37] Bußler S., Rumpold B.A., Jander E., Rawel H.M., Schlüter O.K. (2016). Recovery and techno-functionality of flours and proteins from two edible insect species: Meal worm (*Tenebrio molitor*) and black soldier fly (*Hermetia illucens*) larvae. Heliyon.

[38] Purschke B., Meinlschmidt P., Horn C., Rieder O., Jäger H. (2018). Improvement of techno-functional properties of edible insect protein from migratory locust by enzymatic hydrolysis. Eur. Food Res. Technol..

[39] Rumpold B.A., Schlüter O. (2015). Insect-based protein sources and their potential for human consumption: Nutritional composition and processing. Anim. Front..

[40] Mishyna M., Chen J., Benjamin O. (2019). Sensory attributes of edible insects and insect-based foods–Future outlooks for enhancing consumer appeal. Trends Food Sci. Technol..

[41] Nongonierma A.B., FitzGerald R.J. (2017). Unlocking the biological potential of proteins from edible insects through enzymatic hydrolysis: A review. Innov. Food Sci. Emerg. Technol..

[42] Otvos L. (2000). Antibacterial peptides isolated from insects. J. Pept. Sci. An Off. Publ. Eur. Pept. Soc..

[43] Chernysh S., Kim S., Bekker G., Pleskach V., Filatova N., Anikin V., Platonov V., Bulet P. (2002). Antiviral and antitumor peptides from insects. Proc. Natl. Acad. Sci. USA.

[44] Billaud C., Adrian J. (2003). Louis-Camille Maillard, 1878–1936. Food Rev. Int..

[45] Nursten H.E. (2005). The Maillard Reaction: Chemistry, Biochemistry, and Implications.

[46] Lund M.N., Ray C.A. (2017). Control of Maillard reactions in foods: Strategies and chemical mechanisms. J. Agric. Food Chem..

[47] Jägerstad M., Reuterswärd A.L., Öste R., Dahlqvist A., Grivas S., Olsson K., Nyhammar T. (1983). Creatinine and Maillard Reaction pProducts as Precursors of Mutagenic Compounds Formed in Fried Beef.

[48] Lee K.-G., Shibamoto T. (2002). Toxicology and antioxidant activities of non-enzymatic browning reaction products. Food Rev. Int..

[49] Vhangani L.N., Van Wyk J. (2016). Antioxidant activity of Maillard reaction products (MRPs) in a lipid-rich model system. Food Chem..

[50] Misra N., Koubaa M., Roohinejad S., Juliano P., Alpas H., Inácio R.S., Saraiva J.A., Barba F.J. (2017). Landmarks in the historical development of twenty first century food processing technologies. Food Res. Int..

[51] Corsini L., Castro R., Barroso C.G., Durán-Guerrero E. (2019). Characterization by gas chromatography-olfactometry of the most odour-active compounds in Italian balsamic vinegars with geographical indication. Food Chem..

[52] Shimoda M., Shiratsuchi H., Nakada Y., Wu Y., Osajima Y. (1996). Identification and sensory characterization of volatile flavor compounds in sesame seed oil. J. Agric. Food Chem..

[53] Mori K., Tamada S., Uchida M., Mizumachi N., Tachibana Y., Matsui M. (1978). Synthesis of optically active forms of seudenol, the pheromone of douglas fir beetle. Tetrahedron.

[54] Pittet A.O., Hruza D.E. (1974). Comparative study of flavor properties of thiazole derivatives. J. Agric. Food Chem..

[55] Qin P., Ma T., Wu L., Shan F., Ren G. (2011). Identification of tartary buckwheat tea aroma compounds with gas chromatography-mass spectrometry. J. Food Sci..

[56] López-Galilea I., Fournier N., Cid C., Guichard E. (2006). Changes in headspace volatile concentrations of coffee brews caused by the roasting process and the brewing procedure. J. Agric. Food Chem..

[57] Schieberle P. (1996). Odour-active compounds in moderately roasted sesame. Food Chem..

[58] Cho S., Kays S.J. (2013). Aroma-active compounds of wild rice (*Zizania palustris* L.). Food Res. Int..

[59] López-López A., Sánchez A.H., Cortés-Delgado A., de Castro A., Montaño A. (2018). Relating sensory analysis with SPME-GC-MS data for Spanish-style green table olive aroma profiling. LWT.

[60] Sawamura M., Thi Minh Tu N., Onishi Y., Ogawa E., Choi H.-S. (2004). Characteristic odor components of Citrus reticulata Blanco (Ponkan) cold-pressed oil. Biosci. Biotechnol. Biochem..

[61] Song H., Sawamura M., Ito T., Kawashimo K., Ukeda H. (2000). Quantitative determination and characteristic flavour of Citrus junos (yuzu) peel oil. Flavour Fragr. J..

[62] Pubchem. https://pubchem.ncbi.nlm.nih.gov/.

[63] The Good Scents Company Information System. http://www.thegoodscentscompany.com/index.html.

[64] Spillane W.J. (2006). Optimising Sweet Taste in Foods.

[65] Mortzfeld F., Hashem C., Vrankova K., Winkler M., Rudroff F. (2020). Pyrazines–valuable flavour & fragrance compounds: Biocatalytic synthesis and industrial applications. Authorea Prepr..

[66] Mortzfeld F.B., Hashem C., Vranková K., Winkler M., Rudroff F. (2020). Pyrazines: Synthesis and industrial application of these valuable flavor and fragrance compounds. Biotechnol. J..

[67] EFSA Panel on Nutrition N.F., Allergens F., Turck D., Castenmiller J., De Henauw S., Hirsch-Ernst K.I., Kearney J., Maciuk A., Mangelsdorf I., McArdle H.J. (2021). Safety of dried yellow mealworm (Tenebrio molitor larva) as a novel food pursuant to Regulation (EU) 2015/2283. Efsa J..

[68] Ramos-Elorduy J., Gonzalez E.A., Hernandez A.R., Pino J.M. (2002). Use of Tenebrio molitor (Coleoptera: Tenebrionidae) to recycle organic wastes and as feed for broiler chickens. J. Econ. Entomol..

[69] Jabir M.A.R., Jabir S.A.R., Vikineswary S. (2012). Nutritive potential and utilization of super worm (Zophobas morio) meal in the diet of Nile tilapia (*Oreochromis niloticus*) juvenile. Afr. J. Biotechnol..

[70] Białowiec A., Micuda M., Szumny A., Łyczko J., Koziel J.A. (2018). Quantification of VOC emissions from carbonized refuse-derived fuel using solid-phase microextraction and gas chromatography-mass spectrometry. Molecules.

[71] Łyczko J., Jałoszyński K., Surma M., Masztalerz K., Szumny A. (2019). HS-SPME analysis of true lavender (*Lavandula angustifolia* Mill.) leaves treated by various drying methods. Molecules.

[72] Adams R.P. (2007). Identification of *Essential oil* Components by Gas Chromatography/Mass Spectrometry.

