# Supplementary material for: Sensory and Chemical Characteristic of Two Insect Species: Tenebrio molitor and Zophobas morio Larvae Affected by Roasting Processes
Source: Molecules. 2021 May 4;26(9):2697. doi: 10.3390/molecules26092697 (PMC8124484; doi:10.3390/molecules26092697)
Supplement: Supplementary file 1 [file molecules-26-02697-s001.zip › molecules-1130318-SI.pdf]

Supplementarny materials

Selected chromatograms:

S1:

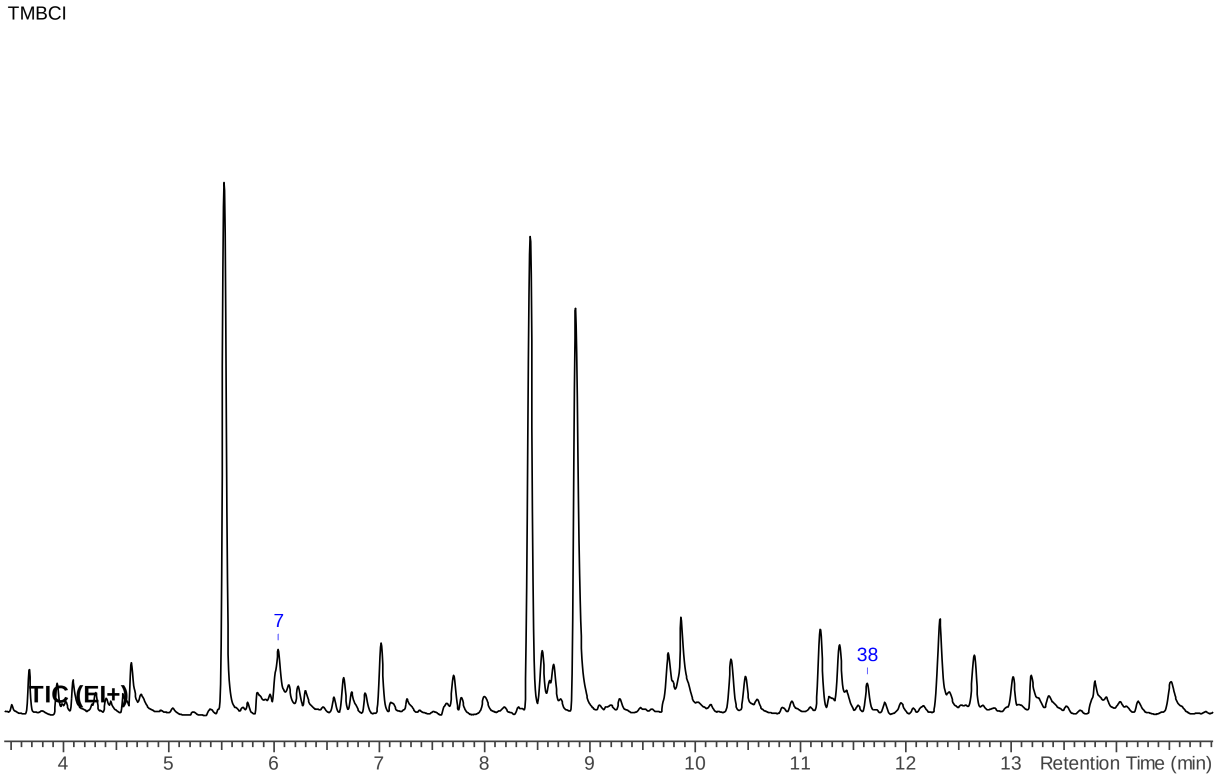

S2:

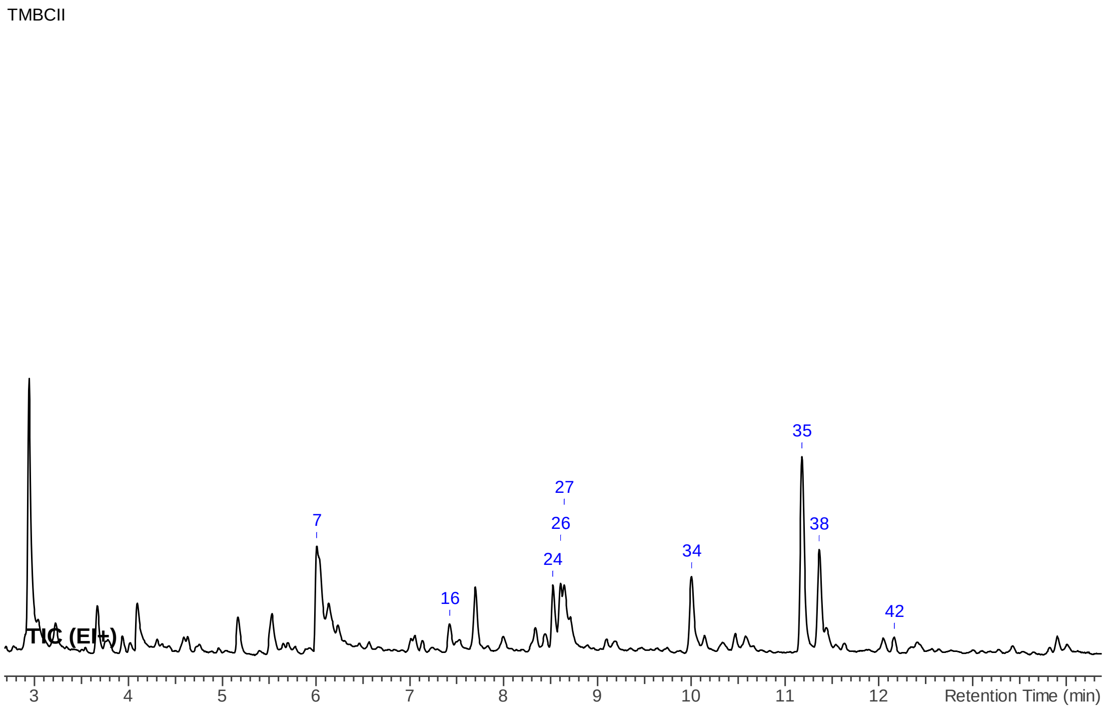

S3:

TMBCIII

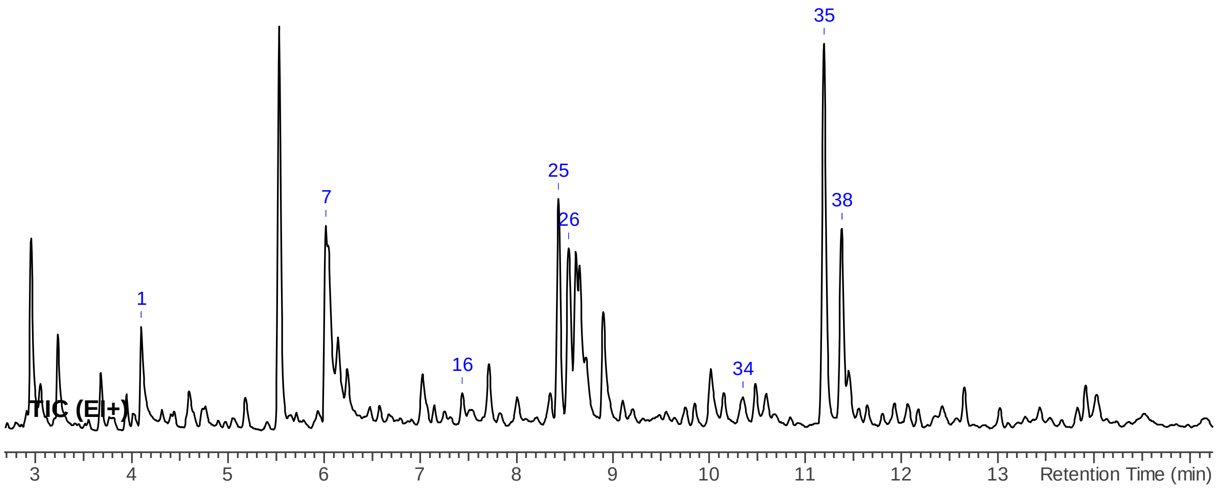

S4:

ZMBCI

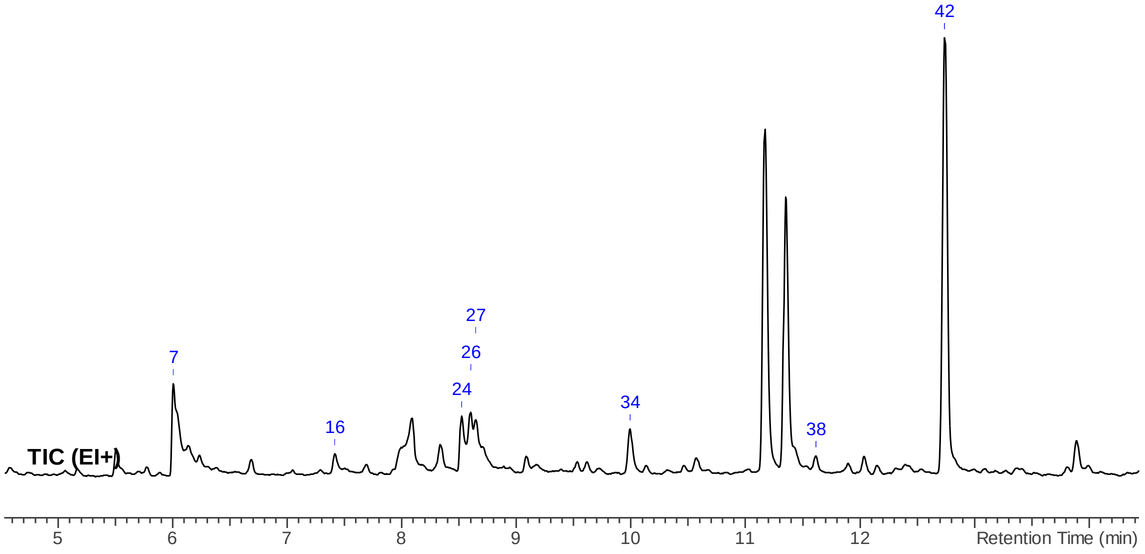

S5:

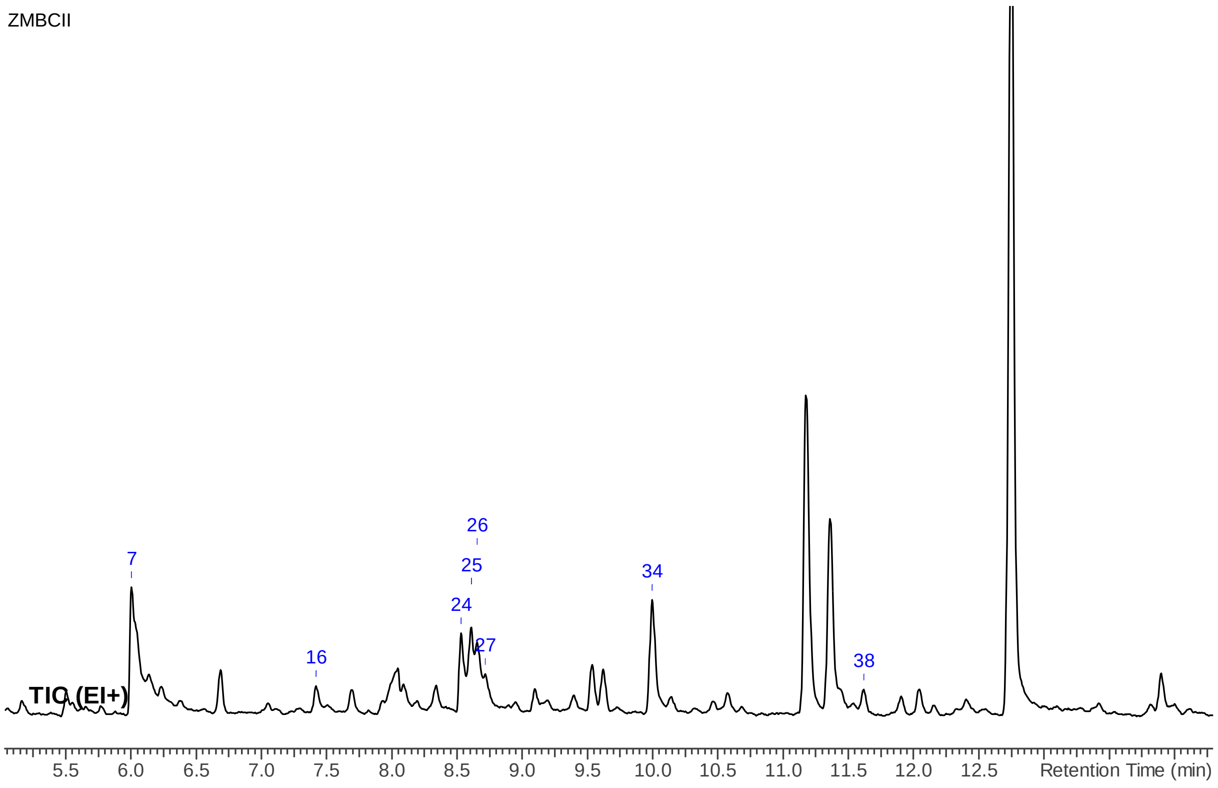
ZMBCII
TIC (EI+)
7
16
24
25
26
27
34
38
5.5
6.0
6.5
7.0
7.5
8.0
8.5
9.0
9.5
10.0
10.5
11.0
11.5
12.0
12.5
Retention Time (min)

S6:

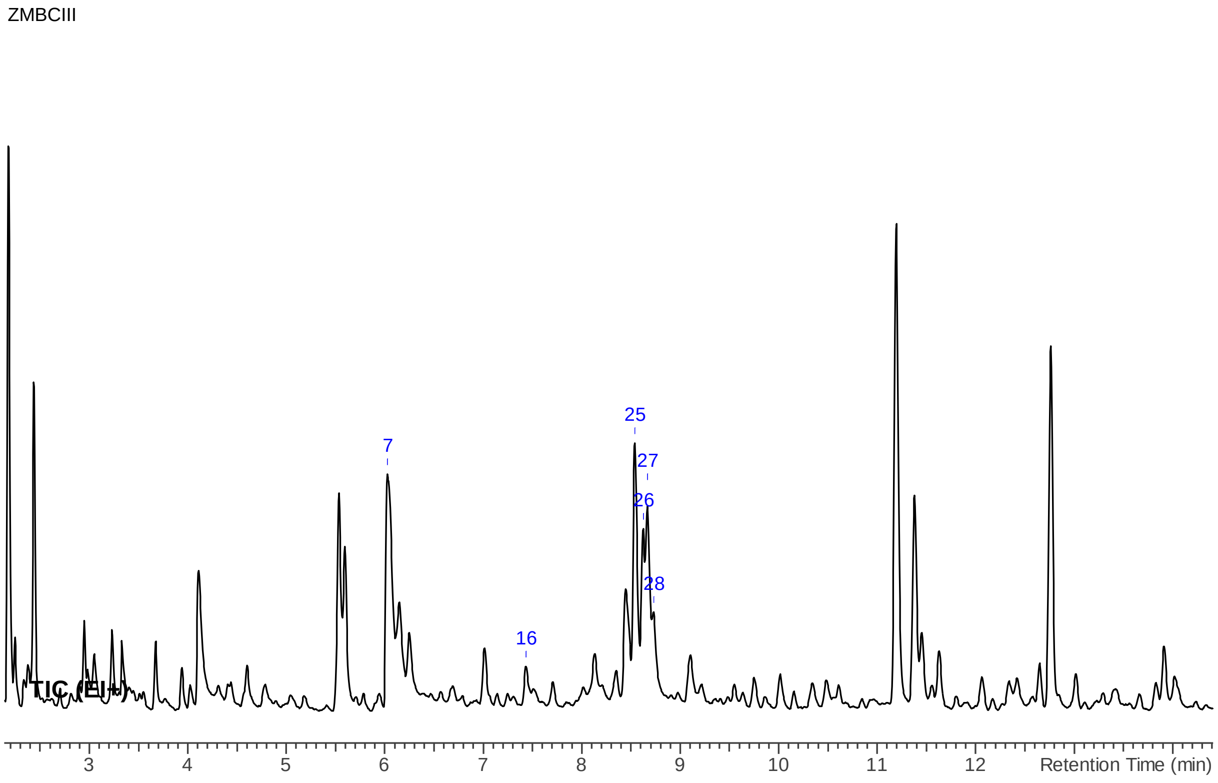
ZMBCIII
TIC (EI+)
7
16
25
27
26
28
3
4
5
6
7
8
9
10
11
12
Retention Time (min)

S7:

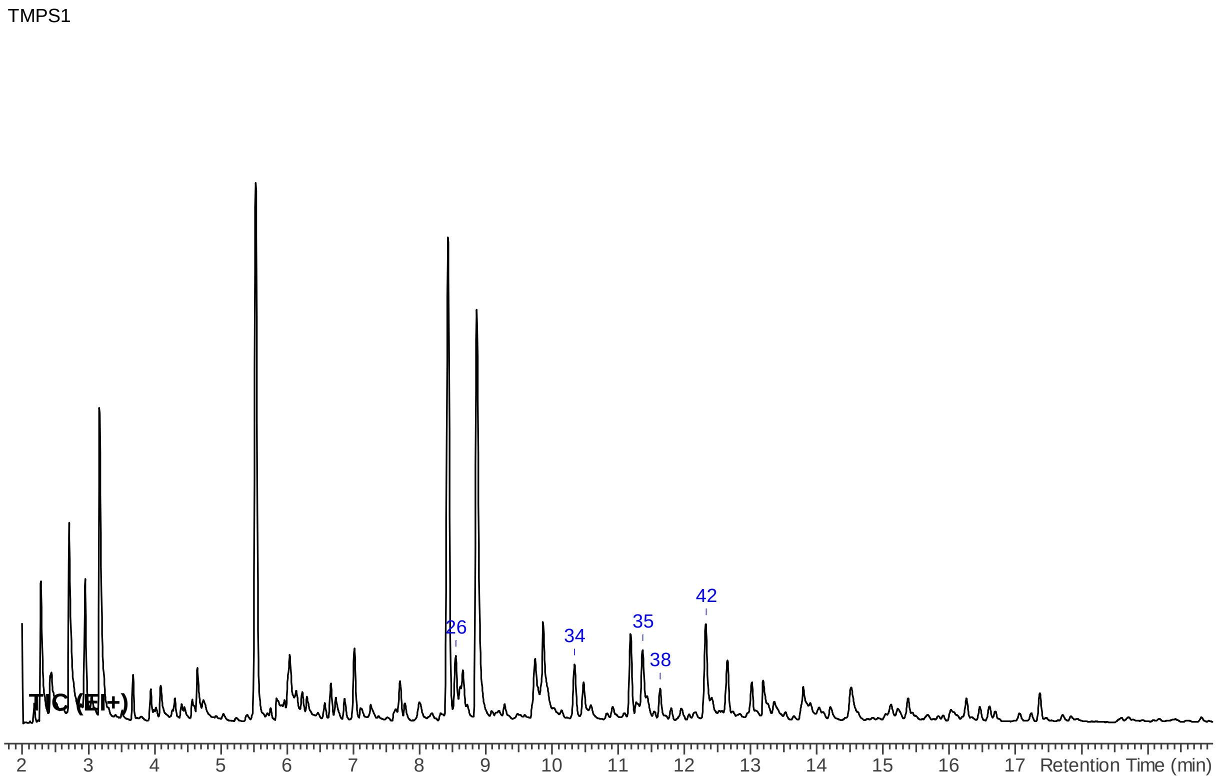
TMPS1
26
34
35
38
42
TIC (EI+)
2
3
4
5
6
7
8
9
10
11
12
13
14
15
16
17
Retention Time (min)

S8:

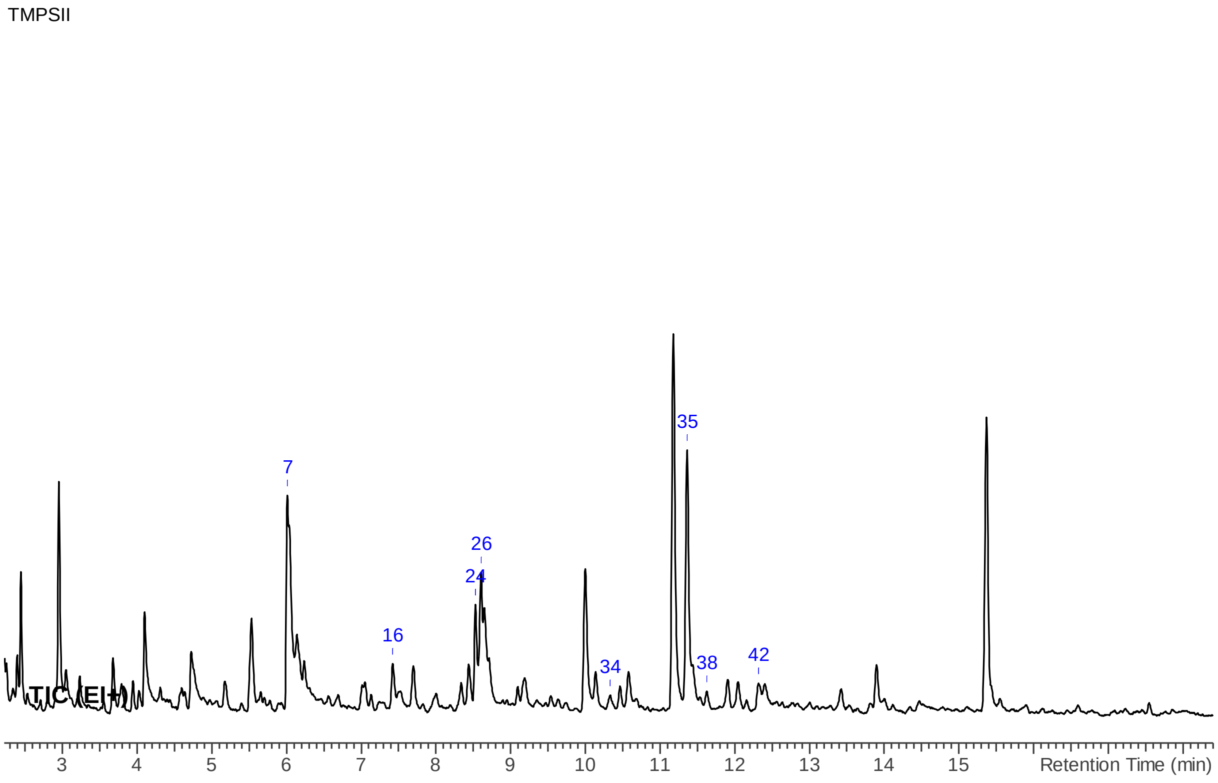
TMPSII
7
16
24
26
34
35
38
42
TIC (EI+)
3
4
5
6
7
8
9
10
11
12
13
14
15
Retention Time (min)

S9:

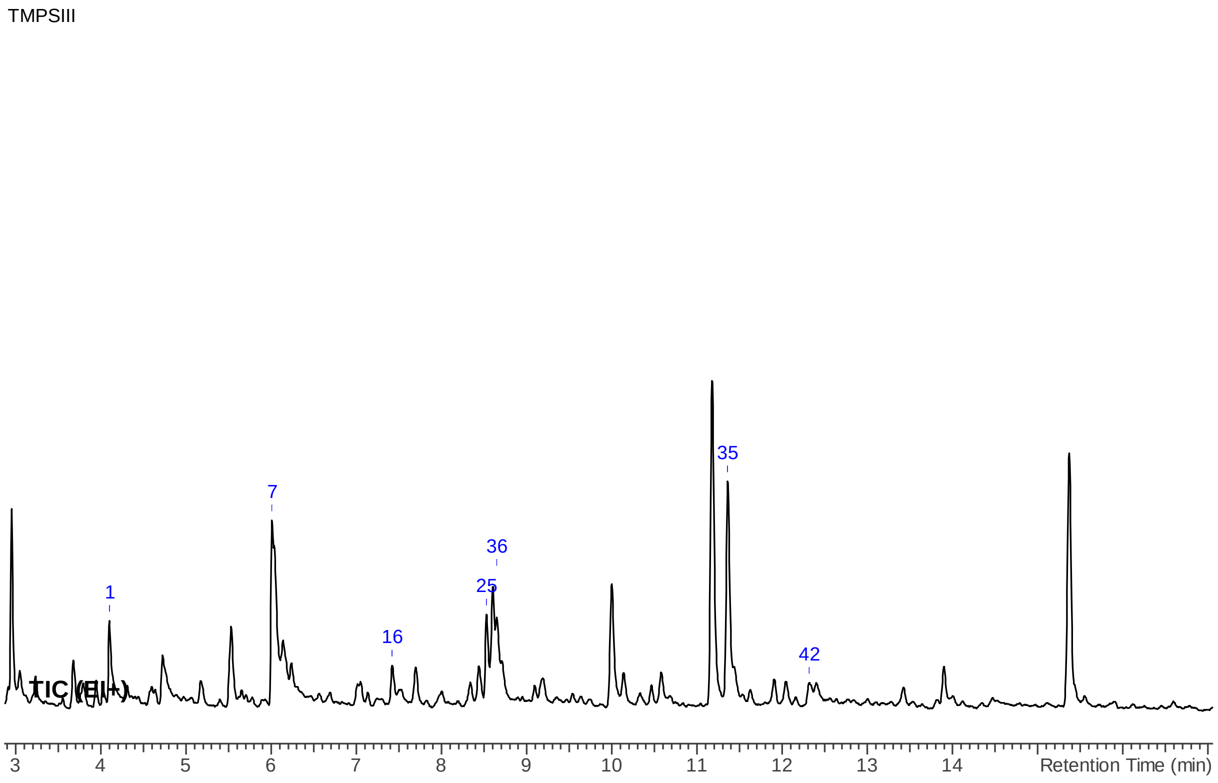
TMPSIII
TIC (EI+)
1
7
16
25
36
35
42
3
4
5
6
7
8
9
10
11
12
13
14
Retention Time (min)

S10:

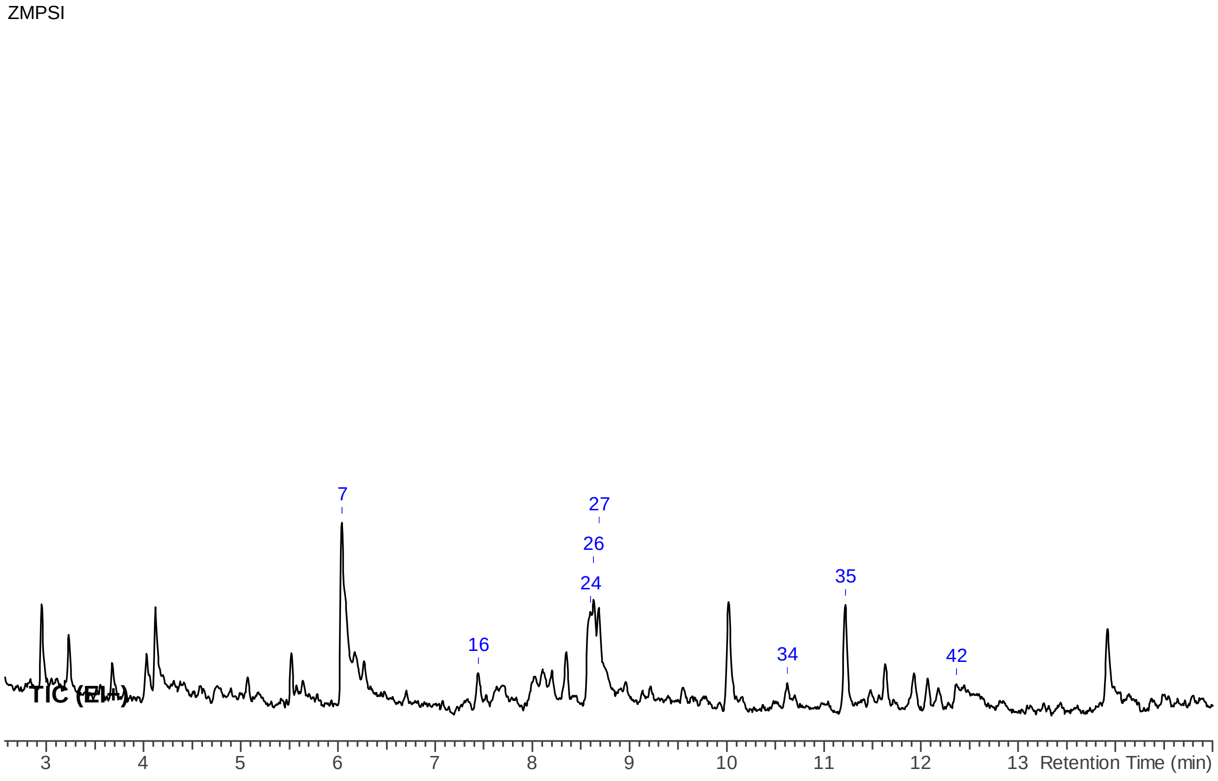
ZMPSI
TIC (EI+)
7
16
24
26
27
34
35
42
3
4
5
6
7
8
9
10
11
12
13
Retention Time (min)

S11:

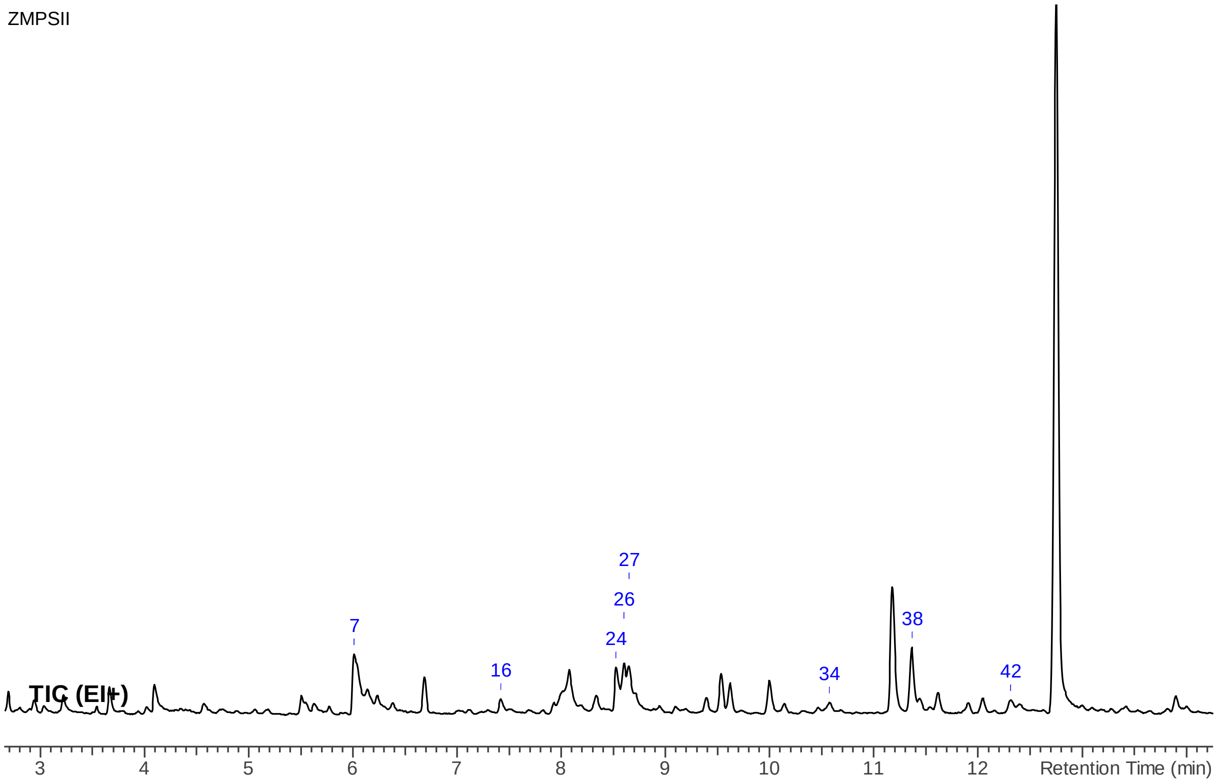
ZMPSII
27
26
24
7
16
34
38
42
TIC (EI+)
3
4
5
6
7
8
9
10
11
12
Retention Time (min)

S12:

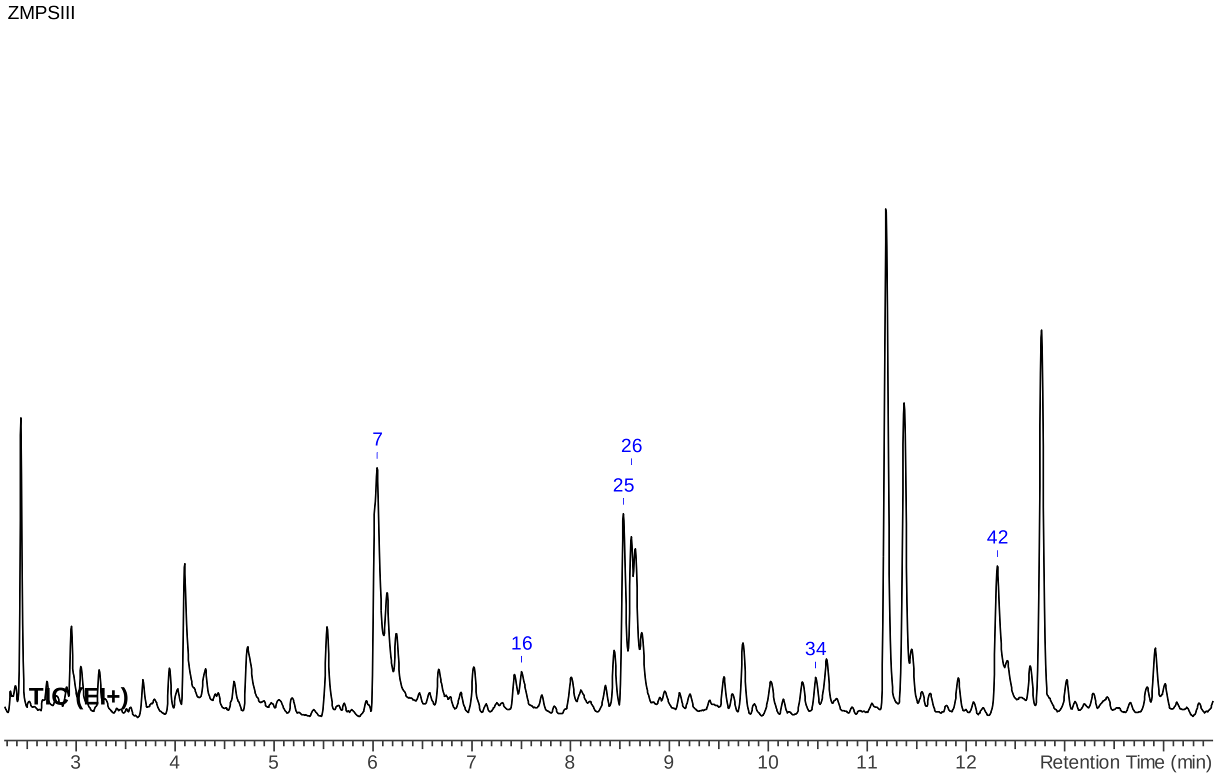
ZMPSIII
7
26
25
42
16
34
TIC (EI+)
3
4
5
6
7
8
9
10
11
12
Retention Time (min)

TABLES:

S13:

Table S1. Concentration and odor activity values of aroma active compounds (OAV) and %OAV of roasted *Tenebrio molitor* larvae.

No	Compound	TMPS I			TMPSII			TMPS III			TMBCI			TMBCII			TMBCIII		
		ppm [µg/g]	OAV	%OAV	ppm [µg/g]	OAV	%OAV	ppm [µg/g]	OAV	%OAV	ppm [µg/g]	OAV	%OAV	ppm [µg/g]	OAV	%OAV	ppm[µg/g]	OAV	%OAV
1	Furan-2-carbaldehyde	n.d.	-	-	n.d.	-	-	0.745±0.096	75.079	4.30	n.d.	-	-	n.d.	-	-	0.055±0.008	11.110	1.65
7	2,5-Dimethylpyrazine	n.d.	-	-	0.044±0.005	0.436	0.14	0.049±0.006	61.491	3.52	0.098±0.007	0.980	69.56	0.437±0.011	4.366	0.20	0.049±0.003	73.491	10.88
16	Benzaldehyde	n.d.	-	-	0.006±0.003	128.446	42.59	0.002±0.001	518.089	29.67	n.d.	-	-	0.076±0.0030	10.591	0.49	0.005±0.003	157.840	23.37
24	2-Ethyl-6-methylpyrazine	n.d.	-	-	0.012±0.003	0.119	0.04	n.d.	-	-	n.d.	-	-	0.167±0.012	1.672	0.08	n.d.	-	-
25	2-Ethyl-5-methylpyrazine	n.d.	-	-	n.d.	-	-	0.058±0.004	0.004	0.00	n.d.	-	-	n.d.	-	-	0.063±0.009	0.004	0.00
26	2,3,5-Trimethylpyrazine	0.031±	0.309	0.01	0.013±0.004	0.132	0.04	0.048±0.003	0.485	0.03	n.d.	-	-	0.189±0.013	1.893	0.09	0.046±0.005	0.456	0.07
27	2-Ethyl-3-methylpyrazine	n.d.	-	-	n.d.	-	-	n.d.	-	-	n.d.	-	-	0.095±0.008	0.789	0.037	n.d.	-	-
34	Isobutylpyrazine	0.054±	3357.173	99.24	0.003±0.002	170.044	56.38	0.017±0.002	1080.254	61.86	n.d.	-	-	0.033±0.007	2092.090	97.22	0.011±0.004	423.158	62.64
35	Oct-2-en-1-ol	0.027±	22.180	0.66	0.003±0.001	2.294	0.76	0.012±0.002	9.999	0.57	n.d.	-	-	0.048±0.006	39.793	1.85	0.011±0.002	9.391	1.39

38	Nonan-2-one	0.025±	0.246	0.01	0.003± 0.002	0.026	0.01	n.d.	-	-	0.043± 0.003	0.429	30.44	0.022± 0.003	0.220	0.01	0.006± 0.003	0.062	0.01
42	Maltol	0.108±	3.094	0.09	0.004± 0.001	0.119	0.04	0.032± 0.004	0.918	0.05	n.d.	-	-	0.022± 0.002	0.614	0.03	n.d.	-	-

S14:

Table S2. Concentration and odor activity values of aroma active compounds (OAV) and %OAV of roasted *Zophobas morio* larvae.

No	Compound	ZMPS I			ZMPS II			ZMPS III			ZMBCI			ZMBCII			ZMBCIII		
		ppm [µg/g]	OAV	%OAV	ppm [µg/g]	OAV	%OAV	ppm [µg/g]	OAV	%OAV	ppm [µg/g]	OAV	%OAV	ppm [µg/g]	OAV	%OAV	ppm[µg/g]	OAV	%OAV
1	Furan-2-carbaldehyde	n.d.	-	-	n.d.	-	-	n.d.	-	-	n.d.	-	-	n.d.	-	-	n.d.	-	-
7	2,5-Dimethylpyrazine	0.011± 0.002	0.112	0.05	0.028± 0.005	0.280	1.35	0.457± 0.037	4.566	6.53	0.030± 0.005	0.298	2.03	0.052± 0.006	0.522	6.01	0.063± 0.027	0.627	11.79
16	Benzaldehyde	0.004± 0.002	0.005	0.002	0.003± 0.001	0.004	0.02	0.006± 0.002	0.008	0.01	0.004± 0.001	0.005	0.03	0.825± 0.073	1.031	11.89	0.655± 0.89	0.819	15.39
24	2-Ethyl-6-methylpyrazine	0.014± 0.001	0.141	0.06	0.012± 0.001	0.119	0.57	n.d.	-	-	0.013± 0.003	0.126	0.85	0.008± 0.001	0.084	0.97	n.d.	-	-
25	2-Ethyl-5-methylpyrazine	n.d.	-	-	n.d.	-	-	0.037± 0.004	0.002	0.003	n.d.	-	-	0.009± 0.003	0.001	0.01	0.047± 0.09	0.003	0.06
26	2,3,5-Trimethylpyrazine	0.021± 0.003	0.205	0.09	0.014± 0.002	0.136	0.65	0.027± 0.011	0.271	0.39	0.013± 0.005	0.126	0.86	0.009± 0.003	0.086	0.99	0.036± 0.007	0.362	6.80
27	2-Ethyl-3-methylpyrazine	0.004± 0.002	0.035	0.02	0.014± 0.003	0.113	0.54	n.d.	-	-	0.002± 0.001	0.021	0.14	0.004± 0.003	0.030	0.35	0.011± 0.002	0.088	1.65

34	Isobutylpyrazine	0.003±0.001	217.343	98.76	0.0003±0.0001	19.969	96.17	0.001±0.0005	64.042	91.60	0.0002±0.0001	13.976	94.95	0.0001±0.00007	6.838	78.83	0.00005±0.00002	3.424	64.32
35	Oct-2-en-1-ol	0.002±0.001	1.602	0.73	n.d.	-	-	n.d.	-	-	n.d.	-	-	n.d.	-	-	n.d.	-	-
38	Nonan-2-one	n.d.	-	-	0.003±0.001	0.031	0.15	n.d.	-	-	0.002±0.001	0.018	0.12	0.008±0.002	0.083	0.95	n.d.	-	-
42	Maltol	0.022±0.004	0.624	0.28	0.004±0.002	0.114	0.55	0.036±0.007	1.025	1.47	0.005±0.002	0.151	1.02	n.d.	-	-	n.d.	-	-

S15:

Table S3. Aroma profile of the roasted at 160 °C *Tenebrio molitor* larvae fed BC or PS.

descriptor	TMBCI	TMPSI
roasted bacon	9.1±0.9a	7.9±0.11b
bread	4.1±0.3a	3.2±0.5b
oily	2.3±0.3a	2.9±0.5b
burnt	3.5±0.4a	4.8±0.6b
malty	5.8±0.7a	5.2±0.7b

Mean values with different letters (a–b) within the same row were statistically different (p = 0.05), the same letters form one homogeneous group. Values expressed as mean ± standard deviation.

S16:

Table S4. Aroma profile of the roasted at 180 °C *Tenebrio molitor* larvae fed BC or PS.

descriptor	TMBCII	TMPSII
roasted bacon	5.2±0.9a	5.3±0.7a
bread	7.8±1.1a	5.2±0.8b
oily	5.1±0.9a	4.3±0.6b
burnt	4.4±0.9b	5.1±0.3a
malty	3.0±0.4a	3.0±0.6a

Mean values with different letters (a–b) within the same row were statistically different (p = 0.05), the same letters form one homogeneous group. Values expressed as mean ± standard deviation.

S17:

Table S5. Aroma profile of the roasted at 200 °C *Tenebrio molitor* larvae fed BC or PS.

descriptor	TMBCIII	TMPSIII
roasted bacon	3.1±0.6a	2.1±0.8a
bread	6.1±0.5a	6.2±0.9a
oily	7.0±0.4a	7.1±1.0a
burnt	7.2±0.9b	8.0±1.0a
malty	3.1±0.4a	2.3±0.5b

Mean values with different letters (a–b) within the same row were statistically different (p = 0.05), the same letters form one homogeneous group. Values expressed as mean ± standard deviation.

S18:

Table S6. Aroma profile of the roasted at 160 °C *Zophobas morio* larvae fed BC or PS.

descriptor	ZMBCI	ZMPSI
roasted bacon	7.1±1.1a	8.2±0.9b
bread	6.2±0.7a	3.2±0.6b
oily	6.1±0.9a	6.3±0.6b
burnt	5.1±1.2a	6.1±03.b
malty	5.2±0.7a	4.2±0.6b

Mean values with different letters (a–b) within the same row were statistically different (p = 0.05), the same letters form one homogeneous group. Values expressed as mean ± standard deviation.

S19

Table S7. Aroma profile of the roasted at 180 °C *Zophobas morio* larvae fed BC or PS.

descriptor	ZMBCII	ZMPSII
roasted bacon	7.2±0.6a	7.1±0.8a
bread	2.4±0.6b	3.1±0.6a
oily	5.1±0.7a	5.1±0.8a
burnt	7.0±1.1a	7.1±0.8a
malty	7.0±0.9a	7.0±1.2a

Mean values with different letters (a–b) within the same row were statistically different (p = 0.05), the same letters form one homogeneous group. Values expressed as mean ± standard deviation.

S20:

Table S8. Aroma profile of the roasted at 200 °C *Zophobas morio* larvae fed BC or PS.

descriptor	ZMBCIII	ZMPSIII
roasted bacon	6.9±0.5a	6.1±0.4b
bread	2.0±0.7a	2.1±0.4a
oily	5.0±0.6a	4.2±0.5b
burnt	8.0±0.7b	9.0±1.3a
malty	4.1±0.7a	3.0±0.7b

Mean values with different letters (a–b) within the same row were statistically different ($p = 0.05$), the same letters form one homogeneous group. Values expressed as mean ± standard deviation.
